# A mutation in *GJA8* (p.P88Q) is associated with “balloon-like” cataract with Y-sutural opacities in a family of Indian origin

**Published:** 2008-06-17

**Authors:** Vanita Vanita, Jai Rup Singh, Daljit Singh, Raymonda Varon, Karl Sperling

**Affiliations:** 1 Centre for Genetic Disorders, Guru Nanak Dev University, Amritsar, India; 2 Dr. Daljit Singh Eye Hospital, Amritsar, India; 3 Institute of Human Genetics, Charitè, University Medicine of Berlin, Berlin, Germany

## Abstract

**Purpose:**

To detect the underlying genetic defect in a family with three members in two generations affected with bilateral congenital cataract.

**Methods:**

Detailed family history and clinical data were recorded. Mutation screening in the candidate genes, αA-crystallin (*CRYAA*), βA1-crystallin (*CRYBA1*), βB2-crystallin (*CRYBB2*), γA–γD-crystallins (*CRYGA, CRYGB*, *CRYGC*, and *CRYGD*), connexin-46 (*GJA3*), and connexin-50 (*GJA8*), was performed by bidirectional sequencing of the amplified products.

**Results:**

Affected individuals had “balloon-like” cataract with prominent Y-sutural opacities. Sequencing of the candidate genes showed a heterozygous c.262C>A change in the gene for connexin 50 (*GJA8*), which is localized at 1q21, that resulted in the replacement of a highly conserved proline by glutamine (p.P88Q). This sequence change was not observed in 96 ethnically matched controls.

**Conclusions:**

We report a p.P88Q mutation in *GJA8* associated with Y-sutural cataract in a family of Indian origin. Mutations of the same codon have previously been described in British families with pulverulent cataract, suggesting that modifying factors may determine the type of cataract.

## Introduction

Congenital cataract is one of the most significant causes of visual impairment and childhood blindness worldwide. The prevalence of non-syndromic congenital cataract is estimated to be 1–6 cases per 10,000 live births [1,2]. It is a clinically and genetically heterogeneous ocular lens disorder. Interfamilial and intrafamilial phenotypic variation is quite significant in congenital cataract, and various types and sub-types have been reported [3-5]. Approximately one-third of the cases show a positive family history [6]. All three Mendelian modes of inheritance exist for congenital cataract. However, an autosomal dominant mode of transmission is reported to be the most common [5]. At least 27 loci have been mapped. The identified genes encode proteins such as crystallins, the major structural lens proteins (CRYAA, CRYAB, CRYBA1/A3, CRYBB1, CRYBB2, CRYBB3, CRYGC, CRYGD, and CRYGS), gap junctional proteins, the connexins (GJA3 and GJA8), major intrinsic protein (MIP/MIP26), lens integral membrane protein 2 (LIM2), beaded filament proteins (BFSP1, BFSP2), and heat shock protein (HSF4) as reviewed by Reddy et al. [2] and Hejtmancik [7].

Pulverulent cataract was the first autosomal disease locus mapped. In 1963, Renwick and Lawler described linkage to the Duffy blood group locus [8], which was found to cosegregate with an uncoiler element of chromosome 1q by Donahue in 1968 [9]. Thirty years later, Shiels analyzed the same cataract family and reported a missense mutation in codon 88 of the connexin 50 (*GJA8*) gene at 1q21 [10], leading to the substitution of proline by serine (p.P88S). Here, we report a cataract family of Indian origin with three affected members in two generations with a proline to glutamine mutation of the same codon (p.P88Q) but with balloon-like cataract with prominent Y-sutural opacities. Now, more than a dozen different mutations in *GJA8* associated with different cataract phenotypes have been identified (Table 1).

**Table 1:** Mutations identified in connexin 50 in association with different cataract phenotypes in different congenital cataract families.

**Amino acid change**	**Location/GJA8 Domain**	**Cataract type**	**Phenotype description**	**Origin of family**	**References**
p.R23T	Cytoplasmic NH_2_-terminal	Congenital nuclear	Progressive, dense nuclear (fetal/embryonal)	Iranian	[23]
p.V44E	First transmembrane domain (M1)	Congenital cataract and microcornea	Total lens opacification	Indian	[24]
p.W45S	First transmembrane domain (M1)	Jellyfish-like cataract and microcornea	Axial lens opacity with finger-like projections extended in all directions	Indian	[25]
p.D47N	First extracellular loop (E1)	Nuclear pulverulent	Opacities confined to the fetal and embryonal nucleus	English	[26]
p.D47Y	First extracellular loop (E1)	Autosomal dominant congenital cataract	Autosomal dominant congenital cataract	Chinese	[27]
p.E48K	First extracellular loop (E1)	Zonular nuclear pulverulent	Non-progressive, fine dust-like opacities, more dense throughout the nucleus. Several cortical riders in the zonular region	Pakistani	[28]
p.V64G	First extracellular loop (E1)	Congenital nuclear	Congenital nuclear cataract	Chinese	[29]
p.V79L	Second transmembrane domain (M2)	Full moon like with Y-sutural opacities	Stationary cataract both Y-sutures affected. No opacities in the embryonal nucleus, fine granular white opacity outside the embryonal nucleus in the fetal nuclear region	Indian	[22]
p.P88Q	Second transmembrane domain (M2)	Lamellar pulverulent	Pulverulent opacities in the fetal nucleus, embryonal nucleus clear	English	[20]
p.P88Q	Second transmembrane domain (M2)	Balloon-like cataract with Y-sutural opacities	Fetal nucleus and Y-sutures affected. In between the Y-sutures, feathery opacities are present. Three riders present at the perimetry of opaque fetal nucleus. No “pulverized” dust-like opacities in the lens	Indian	present study
p.P88S	Second transmembrane domain (M2)	Zonular pulverulent	Non-progressive innumerable powdery opacities located in the nuclear and lamellar zones. Affects both the embryonic and fetal nucleus: “total nuclear cataract”	English	[10]
p.P189L	Second extracellular loop (E2)	Congenital cataract and microcornea	Star shaped nuclear opacity with a whitish central core	Danish	[30]
p.R198Q	Second extracellular loop (E2)	Cataract and microcornea	Posterior subcapsular	Indian	[24]
p.203fs	Second extracellular loop (E2)	Cataract and nystagmus	Total cataract and nystagmus	Indian	[31]
p.I247M	Cytoplasmic COOH-terminus	Zonular pulverulent	Progressive, non homogeneous opacity consisting of opaque particles of different sizes, most of these very small, distributed unequally in a disc of 5 mm in diameter in the center of the lens. Also a slightly cloudy inhomogeneous area of 2 mm at posterior pole	Russian	[32]
p.S276F	Cytoplasmic COOH-terminus	Pulverulent nuclear	White granular opacities in fetal and embryonal nucleus	Chinese	[33]

This table provides information on specific mutations identified in different domains and regions of connexin 50 in association with congenital cataract in families belonging to different ethnic groups. Most of these mutations are missense mutations, and the cataract phenotypes are zonular/nuclear pulverulent type with powdery dust like opacities. However, the phenotype balloon-like cataract with Y-sutural opacities linked with p.P88Q substitution observed in present family had no pulverized dust like opacities in the lens. The feathery opacities in between the Y-sutural opacities and three riders at the perimetry of the opaque fetal nucleus are very much prominent in the affected lenses in the present family.

## Methods

### Family description

**Figure 1:**
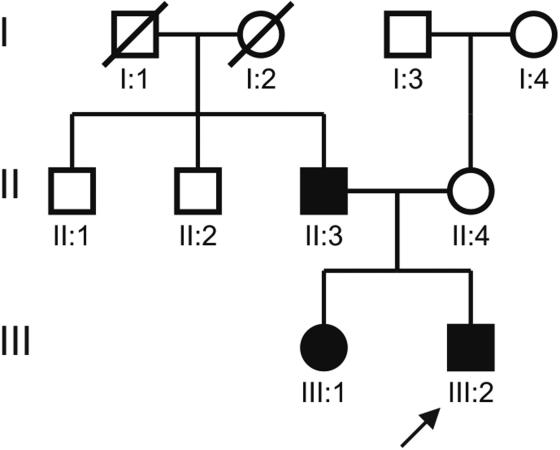
Pedigree of a family with individuals affected by bilateral congenital cataract. The pedigree of an autosomal dominant congenital cataract (ADCC) family shows three members in two generations as affected (filled squares and circle). The proband (III:2), who was diagnosed as bilaterally affected with congenital cataract at the age of seven years, is indicated with an arrow. His elder sister (III:1) was diagnosed with bilateral cataract when she was six years old. The affected father (II:3) of these children underwent cataract extraction in both of his eyes in the first decade of his life.

The proband, a seven-year-old male child, was diagnosed with bilateral cataract. The family history revealed three affected members in two generations (Figure 1). The ophthalmologic examination, including slit lamp examination, was performed on a total of four members of this family; the father (Figure 1; II:3), who had a history of cataract extraction in childhood, and two of his bilaterally affected children (Figure 1; III:1 and III:2). The proband’s mother (Figure 1; II:4) was diagnosed as unaffected.

### Mutation analysis

Informed consent was obtained for each individual studied. This study was approved by the ethics review board of the Guru Nanak Dev University (Amritsar, Punjab, India), consistent with the provisions of the Declaration of Helsinki. Blood was drawn and DNA isolated using method of Adeli and Ogbonna [11]. Mutation screening was performed in the exonic regions of the following candidate genes: *CRYAA* (21q22.3; GenBank NM_000394), *CRYBA1* (17q11-q12; GenBank NM_005208), *CRYBB2* (22q11.2; GenBank NM_000496), *CRYGA* (2q33-q35; GenBank NM_014617), *CRYGB* (2q33-q35; GenBank NM_005210), *CRYGC* (2q33-q35; GenBank NM_020989), *CRYGD* (2q33-q35; GenBank NM_006891), *GJA3* (13q11-q13; GenBank NM_021954), and *GJA8* (1q21-q25; GenBank NM_005267). The coding regions and exon-intron boundaries of the candidate genes were amplified using previously published primer sequences [10,12-16]. Genomic DNA from all three affected and one unaffected individual was amplified. Purified polymerase chain reaction (PCR) products were sequenced bidirectionally with ABI BigDye^TM^ Terminator Cycle Sequencing Ready Reaction Kit version 3.1 (Applied Biosystems, Foster City, CA) for a 10 µl final volume containing 5.0 µl of purified PCR product, 4.0 µl of BigDye Terminator ready reaction mix, and 3.2 pmol of primer. Cycling conditions were: 95 °C for 2 min, 25 cycles at 95 °C for 30 s, 52 °C for 15 s, and 60 °C for 4 min. The sequencing reaction products were purified by the isopropanol precipitation method (ABI protocol; Applied Biosystems), resuspended in 10 µl of loading buffer (5:1 ratio of deionized formamide and 25 mM EDTA with blue dextran [50 mg/ml]), denatured at 95 °C for 5 min, and electrophoresed on 4% denaturing polyacrylamide gels on the DNA sequencer (ABI-Prism 377; Applied Biosystems). Sequencing results were assembled and analyzed using the SeqMan II program of the Lasergene package (DNA STAR Inc., Madison, WI).

## Results

### Phenotype description

**Figure 2:**
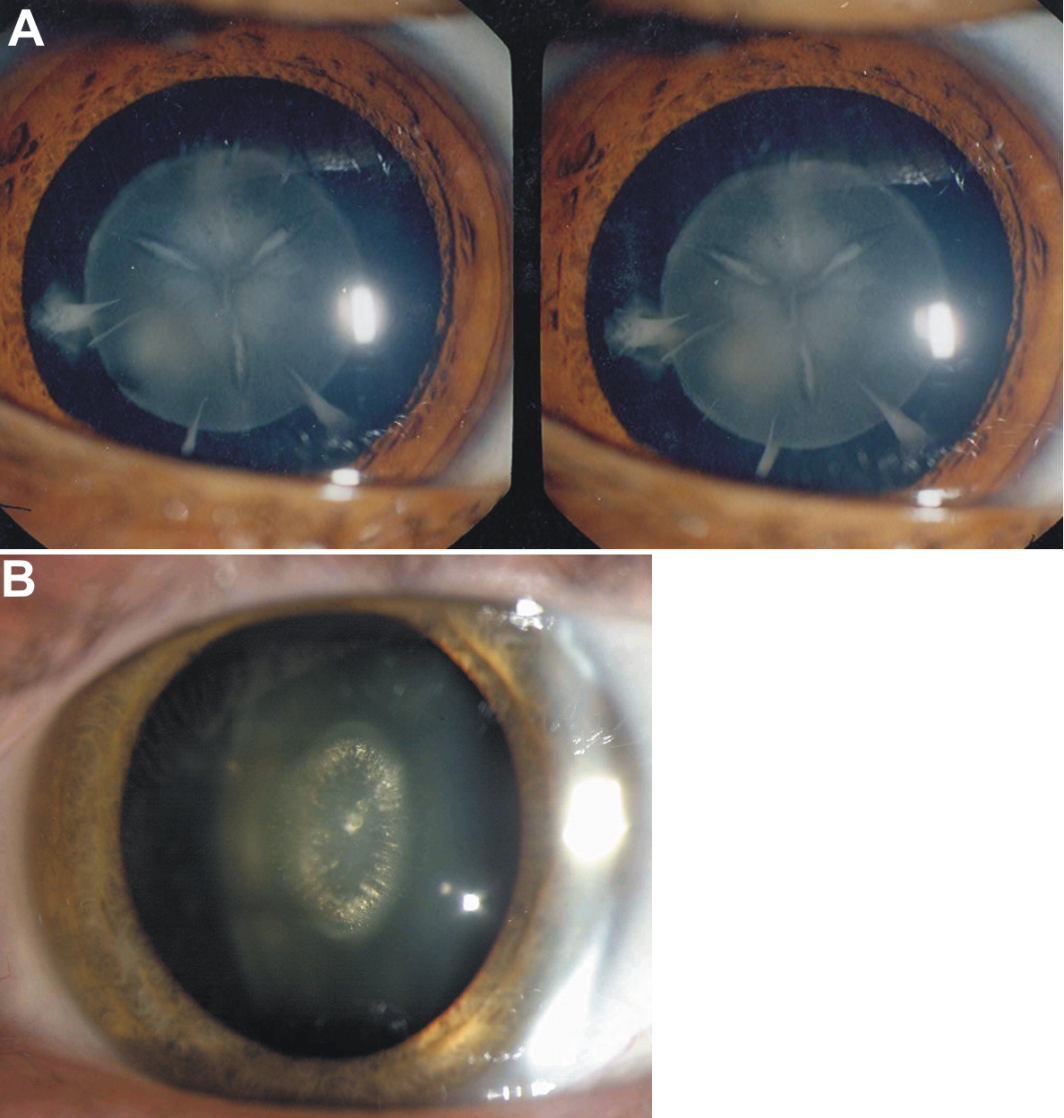
Photograph of cataract phenotypes observed in the present Indian family and in the British family having the identical mutation (p.P88Q) in *GJA8*. **A**: The slit lamp photograph (three-dimensional lens) of a patient shows very prominent feather-like sutural opacities. Apart from sutural opacities, riders are prominent. The inside of the semi-opaque fetal nucleus appeared optically empty. **B**: The photograph from the affected lens of an individual from the British autosomal dominant congenital cataract family with zonular pulverulent cataract [20] shows linear dense vertical opacities inside the fetal nucleus with embryonic nucleus remaining clear and without sutural opacities.

Slit lamp examination of the lenses in affected individuals (III:1, III:2) showed that the cataract affected the fetal nucleus with additional changes on its surface. The fetal nucleus appeared semi-opaque. The Y-sutures on the anterior surface seem sharp and narrow (Figure 2A). In between the Y-sutures, there appeared feathery opacities extending up to three-fourths of the length of the Y-sutures. Along the perimeter of the fetal nucleus, three prominent riders were present. The posterior Y-sutures were not clearly seen through the anterior central feathery opacification, but its presence is in no doubt. Inside the semi-opaque fetal nucleus, there appeared no opacities.

### Mutation screening

**Figure 3:**
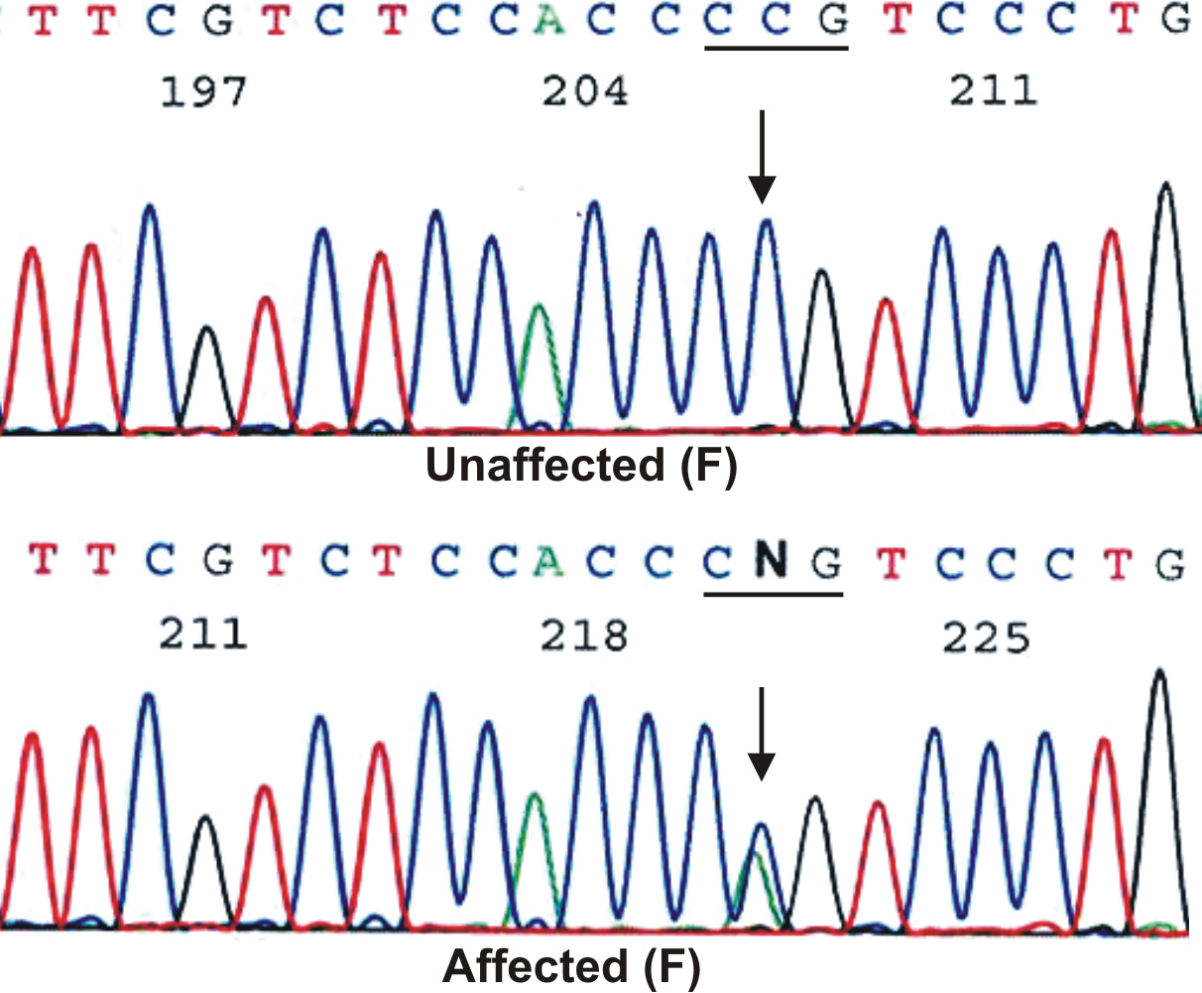
Partial DNA sequence of *GJA8* from an unaffected and an affected individual. The wild type C in the sequence of the unaffected individual and the heterozygous c.262C>A change, which results in the substitution of proline by glutamine at amino acid position 88 (p.P88Q), in the affected individual are indicated by arrows. F=forward strand.

Bidirectional sequencing of the coding regions of the candidate genes showed a heterozygous change, C>A (Figure 3), at position 262 (c.262 C>A) in *GJA8* in all three affected individuals. This substitution was not seen in the unaffected mother or in 96 unrelated control subjects (192 chromosomes) from the same North Indian population as tested by bidirectional sequence analysis (data not shown). The substitution replaces an evolutionarily highly conserved proline by glutamine at amino acid position 88 (p.P88Q) in the second α-helical transmembrane domain 2 (M2) of connexin 50.

## Discussion

Connexins are integral membrane proteins with four transmembrane domains, two extracellular loops, and an intracellular loop with both NH_2_- and COOH-termini localized in the cytoplasm. In humans, at least 20 connexins classified into three families are known [17,18]. Connexin 46 and connexin 50 are responsible for joining the lens cells into a functional syncytium. In addition, connexin 50 is also important for lens growth [19]. The observed p.P88Q substitution is centrally located within the second α-helical transmembrane domain (M2) of connexin 50 and replaces the highly conserved hydrophobic proline by the non-polar glutamine at position 88, in association with the congenital cataract in the present family.

The same mutation was seen in a British family. However, this mutation was associated with lamellar pulverulent cataract [20]. In another family of English descent, a mutation of the same codon lead to the substitution of proline by serine (p.P88S), which is associated with nuclear pulverulent cataract, characterized by innumerable powdery opacities located in the nuclear (central) and perinuclear (lamellar) zones of the lens [8,10]. Further, functional analyses revealed that mutants p.P88S and p.P88Q act as dominant negative inhibitors and significantly decreases the activity of co-expressed wild type connexin 50 [20,21].

Mutations in connexin 46 and connexin 50 have been reported to be linked with cataractogenesis in humans as well as in mice. In humans, over a dozen mutations in each connexin 46 and connexin 50 gene have so far been detected in association with congenital cataract [2,7] and significant interfamilial phenotypic variability. The phenotype in most cases with mutations in connexin 46 and connexin 50 has been described as zonular/nuclear pulverulent cataract [3,5,7]. The cataract phenotype in the present family differs from these as no “pulverized” dust-like opacities are seen in the lens (Table 1). It also differs from the British family with the identical mutation [20], which showed linear dense vertical opacities inside the fetal nucleus with the embryonic nucleus remaining clear and without sutural opacities (Figure 2B). In the present family, Y-sutural opacities are very prominent, comparable with the p.V79L mutation in the second transmembrane domain (M2) of connexin 50, which is linked with “full moon” like cataract with Y-sutural opacities (Table 1) in another Indian family having 15 affected members in three generations reported previously by us [22].

In summary, we describe a heterozygous p.P88Q mutation in connexin 50 that showed marked phenotypic differences to previously reported cases affecting the same codon. Thus, variants in other genes might act as modifiers of the cataract phenotype.
